# Structure of the Gut and Ovary, with Associated Microbiota Across Life Stages in the Striped Stem Borer *Chilo suppressalis* (Lepidoptera: Crambidae)

**DOI:** 10.3390/insects17070682

**Published:** 2026-06-30

**Authors:** Haiying Zhong, Fang Li, Kaili Yu, Juefeng Zhang

**Affiliations:** Institute of Plant Protection and Microbiology, Zhejiang Academy of Agricultural Sciences, Hangzhou 310021, Zhejiang, China; zhy8085@163.com (H.Z.);

**Keywords:** gut, reproductive system, morphology, ultrastructure, microbiota, stem borer

## Abstract

The striped stem borer *Chilo suppressalis* (Lepidoptera: Crambidae) is one of the most serious pests of water bamboo *Zizania latifolia*. Microbiota dynamics across the life cycle of *C. suppressalis* are a prerequisite for comprehending the symbiotic relationship between *C. suppressalis* and its microbiota. In this study, we characterized the structural features of the gut and ovary, as well as the associated microbiota of *C. suppressalis*. The gut is anatomically divided into the foregut, midgut and hindgut. Notable structural and ultrastructural differences were observed between adults and larvae, the details of which have not been previously documented. Microbial sequencing of gut, ovary, and egg samples revealed variations in relative sequence abundances among these tissues. At the phylum level, Proteobacteria and Firmicutes were the predominant groups. At the family level, Bacillaceae, Enterobacteriaceae, Enterococcaceae, Halomonadaceae, Moraxellaceae, and Streptococcaceae were detected in the gut, ovary, and egg, albeit with different relative sequence abundances. The larval midgut exhibited the highest bacteria diversity among all samples examined. The compositional distribution of bacterial genera varied considerably across developmental stages, diet, and gut compartment, and some genera were identified as core microbial taxa. These findings provide a descriptive account of the microbial community structure associated with different tissues and life stages of *C. suppressalis*. They also provided important insights into the investigation of insect–bacteria symbioses, thereby facilitating effective biocontrol of this species.

## Simple Summary

The striped stem borer (*Chilo suppressalis*) is a serious pest of water bamboo *Zizania latifolia*. In this study, we characterized the structural features of the gut and ovary, as well as the associated microbiota in *C. suppressalis*. We described differences in the structure and ultrastructure of larval and adult guts, alongside variations in their associated microbial communities. This work provides a descriptive account of the microbial community structure associated with different tissues and life stages of *C. suppressalis*. These findings not only offer important information that may support future studies on insect–microbe associations but are also relevant to the development of biological control strategies for this pest.

## 1. Introduction

The insect gut is generally divided into three parts: the midgut and hindgut are two of the major regions; nutrients are absorbed in the midgut, while partial nutrients and water are absorbed in the hindgut. Variations in gut structure among different species can be attributed to differences in feeding biology and food types. Solid-food-feeding species usually possess relatively short, thick guts, whereas fluid-feeding species generally have long, narrow guts with some modified structures [1].

The gut is a desirable, nutrient-rich ecological niche where multiple microbiota flourish and reproduce. The midgut is the main site haboring various microbiota, while the anterior hindgut is the most densely symbiont-inhabited site due to the availability of partially digested food from the midgut. In turn, these microbiota may help their hosts utilize life stage-specific resources by providing various functions related to nutrient supplementation [2,3,4], detoxification [5,6], reproduction [7], and population differentiation [8,9]. This is especially evident in phytophagous insects. Gut microbes can be transmitted from one generation to the next, a process known as vertical transmission or inheritance [10,11]. Vertical transmission provides a direct way to establish a gut microbiome [12,13]. However, the gut microbiota may also partially assemble from food and local environments in each generation [3].

Water bamboo *Zizania latifolia* (Gramineae: Oryzeae) is an asexual aquatic vegetable characterized by an edible swollen, plump fleshy stem formed by infection of its meristems by the fungus *Ustilago esculenta*. The striped stem borer (SSB), *Chilo suppressalis*, is one of the destructive generalists of rice *Oryza sativa* in Asia, southern Europe, and northern Africa [14,15,16]. The intercropping pattern of *O. sativa* and *Z. latifolia* facilitates the transfer of *C. suppressalis* from *O. sativa* plants to *Z. latifolia* plants. Compared to those feeding on *O. sativa* plants, *C. suppressalis* feeding on fruit pulps of *Z*. *latifolia* possess higher survival rate, greater pupal weight, and shorter developmental duration [17,18], thus making *C. suppressalis* a serious pest of *Z. latifolia*. The larvae of this species bore into the sheaths, stems, and pulps of *Z. latifolia*, resulting in ‘dead hearts’, ‘dead sheaths’, and ‘boring pulps’ [19,20]. These impacts emphasize the urgent need to develop innovative and effective biological strategies against this pest.

Diet shift can lead to variations in the microbiota throughout the developmental stages of a single host [21]. The larvae of *C. suppressalis* chew solid plant tissues, whereas the adults suck nectar or fruit juice. Nutritional and chemical differences between the larval and adult diets may significantly affect the microbiota residing at different life stages. Characterizing the structure of the microbiota is a prerequisite for its functional roles in the gut. To date, several studies have focused on the effects of resistance on gut microbiota [22,23] or the influence of diet on the larval gut microbiota of *C. suppressalis* [9,24]. However, the structure of the gut and ovary, as well as the associated microbiota across the entire life history of *C. suppressalis*, remains unavailable.

In this study, we characterized the gut and ovary morphology and profiled the gut microbiota of different life stages (larvae, adults, ovaries, and eggs) of *C. suppressalis*, using light microscopy, electron microscopy, transmission electron microscopy, and high-throughput pyrosequencing of microbiota 16S rRNA gene fragments. Our data will be informative to better understand the functional roles of these organs and associated community shifts that occur during life stage transitions and in response to different dietary regimes. These findings may inform novel pest control strategies targeting the sources and transmission routes of gut microbiota.

## 2. Materials and Methods

### 2.1. Plant Materials and Field Cultivation Management

#### 2.1.1. Source of *Zizania latifolia* Seedlings

Ten clusters of the *Z*. *latifolia* variety ‘Zhejiao No. 7’ were obtained from the farmer Jinhai Yao in Zhejiang Province. The farmer had been cultivating *Z*. *latifolia* for 20 years and maintained a long-term technical collaboration with our team. No collection permit was acquired for the obtained plant materials, as the sampling was permitted by the breeder.

#### 2.1.2. Field Domestication and Daily Field Management

All *Z*. *latifolia* clusters were transplanted into an open-air experimental field attached to our laboratory for adaptive cultivation prior to subsequent investigations. Routine field maintenance was performed as follows: regular irrigation was applied to maintain a water layer of 3~5 cm; compound fertilizer was top-dressed twice per growth month; and manual weeding was conducted every 7 days to eliminate competing weeds.

#### 2.1.3. Fresh Pulp Preparation for Feeding Test Insects

Fresh fleshy stem pulps of *Z*. *latifolia* were randomly harvested from cultivated field plots. Damaged, diseased, or aging tissues were discarded, and intact pulps were cleaned with sterile distilled water prior to feeding larvae. All plants materials were utilized following the institutional laboratory management specifications.

#### 2.1.4. Insect Rearing and Colony Establishment

Larvae of *C. suppressalis* were collected from naturally infested stalks of *Z. latifolia* in a cultivated field in Lishui, Zhejiang, where large areas of *Z. latifolia* are exclusively planted. Diseased, injured, and parasitized larvae were discarded prior to rearing. The remaining larvae were transferred into canned bottles (diameter: 10.0 cm; height: 7.0 cm) containing fresh pulps of *Z. latifolia* for rearing. The bottles were incubated at 28 ± 1 °C with a photoperiod of 16 h: 8 h (light/dark) and relative humidity > 80%.

Newly hatched larvae were introduced into canned bottles (diameter: 10.0 cm; height: 7.0 cm) supplied with fresh pulps of *Z. latifolia* and incubated under the same conditions described above. Residual *Z. latifolia* were replaced with fresh pulps every three days. This procedure was repeated until the larvae reached the pupal stage. Pupae were collected and placed into clean petri dishes (8.8 cm in diameter) containing a small piece of moistened sterile sponge to maintain humidity and were incubated under identical environmental conditions.

Approximately six days later, newly emerged females and males were paired in a 1:1 ratio for oviposition and were provided with 8.5% (*w*/*v*) sterile sucrose water as a nutritional supplement. The *C. suppressalis* colony was reared for three consecutive generations before sampling.

### 2.2. Light Microscopy

Females and 4th-instar larvae of *C. suppressalis* were anesthetized prior to dissection. Under a Motic SMZ168 Stereoscopic Zoom Microscope (Motic China Group Co., Ltd., Xiamen, China). The phosphate-buffered saline (PBS) was formulated by mixing sodium dihydrogen phosphate solution (Sangon Biotech (Shanghai) Co., Ltd., Shanghai, China) and disodium hydrogen phosphate solution (Sangon Biotech (Shanghai) Co., Ltd., Shanghai, China) in a certain proportion. The guts and reproductive systems of females, as well as the guts of larvae, were dissected out in phosphate-buffered saline (PBS, 0.2 M, pH 7.2). The dissected tissues were carefully transferred into separate concave dishes, and each part of the organs was observed. Relevant photographs were taken using a Scientific Digital Micrography System (SDMS) (Gatan, Inc., Pleasanton, CA, USA), which was equipped with an Auto-montage imaging system (Gatan, Inc., Pleasanton, CA, USA) and a highly sensitive Qimaging Retiga 2000R digital camera (QImaging, Burnaby, BC, Canada).

### 2.3. Transmission Electron Microscopy (TEM)

Adults and 4th-instar larvae of *C. suppressalis* were anesthetized prior to dissection for collecting their guts and ovaries. Each part of the dissected guts and ovaries was placed in a centrifuge tube with 5.0 mL of 2.5% glutaraldehyde (Thermo Fisher Scientific Inc., Waltham, MA, USA) for prefixation for 12 h at 4 °C. Then, they were rinsed three times in phosphate buffer (PBS, 0.1 M, pH 7.2) and post-fixed in 1% osmium tetroxide (Electron Microscopy Sciences, Hatfield, PA, USA) (PBS, 0.1 M, pH 7.2). After three rinses in the same phosphate buffer (0.1 M, pH 7.2), each part of the guts and ovaries was dehydrated in graded ethanol (30-100%, *v*/*v*) and embedded in Epon 812 (Beijing Zhongjingkeyi Technology Co., Ltd., Beijing, China) [1]. Ultra-thin sections were stained with uranyl acetate (Electron Microscopy Sciences, Hatfield, USA) and lead citrate (Electron Microscopy Sciences, Hatfield, USA), observed under a transmission electron microscope (JEM-1230; JEOL, Tokyo, Japan) at 80 kV, and photographed.

### 2.4. Gut, Ovary and Egg Sample Collection

Healthy, uniformly developed eggs, adults, and 4th-instar larvae of the same batch of *C. suppressalis* were collected. They were externally sterilized with 75% ethanol and rinsed 3 times with sterilized water. For the 50 gut samples from the adults and 4th-instar larvae, the specimens were anesthetized by placing them on ice. The guts and ovaries of adults, as well as the guts of 4th-instar larvae, were dissected using sterilized fine-tip forceps under a Motic SMZ168 Stereoscopic Zoom Microscope in 0.01 M PBS buffer solution (pH = 7.4). The dissected tissues were collected in 1.5 mL centrifuge tubes. These samples were immediately frozen in liquid nitrogen and stored in a −80 °C refrigerator. Additionally, 100 eggs were collected in a 1.5 mL centrifuge tube. In total, six groups of samples were established: larval midguts, larval hindguts, adult midguts, adult hindguts, ovaries, and eggs. Each group of samples was replicated three times.

### 2.5. DNA Isolation, 16S rDNA Amplification and Sequencing

Total DNA was extracted from six groups of samples (guts, ovaries, and eggs) using a Soil DNA Kit (Omega Bio-tek, Norcross, GA, USA) following the manufacturer’s instructions. The purity and concentration of the extracted DNA were measured using a NanoDrop 2000 spectrophotometer (Nanodrop Technologies, Wilmington, DE, USA) [9]. The variable V3-V4 regions of the bacterial 16S rRNA were amplified using the universal primers 341F (5′-CCTAYGGGRBGCASCAG-3′) and 806R (5′-GGACTACNNGGGTATCTAAT-3′). The Polymerase chain reaction (PCR) mixture consisted of 4.0 μL 5× FastPfu Buffer (TransGen Biotech Co., Ltd., Beijing, China), 2.0 μL of 2.5 mM dNTPs (TransGen Biotech Co., Ltd., Beijing, China), 0.4 μL of FastPfu Polymerase (TransGen Biotech Co., Ltd., Beijing, China), 0.8 μL of each primer (5.0 μM), and 10 ng of template DNA. The amplification procedure included an initial denaturation at 95 °C for 2 min, followed by 25 cycles of denaturation at 95 °C for 30 s, annealing at 50 °C for 30 s, and extension at 72 °C for 30 s, with a final extension at 72 °C for 5 min [9]. The concentration of the PCR products was examined using 2% agarose gel electrophoresis and quantified via a NanoDrop 2000 (Thermo Fisher Scientific (Thermo Scientific), Wilmington, DE, USA). Purified amplicons were pooled in equimolar and subjected to paired-end sequencing (2 × 250 bp) on an Illumina MiSeq6000 platform (Illumina, Inc., San Diego, CA, USA).

### 2.6. Processing of Sequencing Data

#### 2.6.1. Raw Data Quality Control

In order to obtain more accurate and reliable results in subsequent bioinformatics analysis, the raw data were pre-processed to obtain clean data by three steps as follows [9]: (1) raw reads were firstly filtered by Trimmomatic v0.33, and the primer sequences were identified and removed by cutadapt 1.9.1 and a custom Perl script, which finally generated high-quality reads without primer sequences; (2) based on overlapping sequences, high-quality reads were assembled by FLASH v1.2.7 for generating clean reads; and (3) chimeric sequences were identified and removed by UCHIME v4.2, generating effective reads.

#### 2.6.2. OTU Analysis and Species Annotation

Picking the representative sequences as proxies of a species is a key step in amplicon analysis. The representative sequence selection was clustered to operational taxonomic units (OTUs). Sequences with ≥97% similarity were assigned to the same OTUs using USEARCH (version 10, http://drive5.com/uparse/, accessed on 16 May 2024). The representative sequence for each OTU was annotated with a threshold of 0.8 using UCLUST v1.2.22q by searching the SILVA database (silva 128, http://www.arb-silva.de, accessed on 16 May 2024). For comparisons between samples, the OTU abundances were normalized by the number obtained from the sample with the lowest counts.

#### 2.6.3. Alpha Diversity Analysis

Alpha diversity reflects richness and diversity of species within a single sample, including richness and evenness measurements. For each sample, alpha diversity metrics (i.e., Chao1, Shannon and Coverage) were evaluated by Mothur (version 1.30.1, https://mothur.org/wiki/, accessed on 16 May 2024). Alpha diversity indices were compared among samples using the Tukey method (*p* = 0.05) with R software (Version 3.4.4 https://www.r-project.org/, accessed on 16 May 2024).

Non-metric multidimensional scaling (NMDS) is a nonlinear dimensionality reduction ordination method based on a distance matrix. Its core objective is to preserve the rank order of original distances between samples in a low-dimensional space, rather than their absolute numerical values; it is particularly suitable for datasets with nonlinear relationships or complex data distributions, such as those in ecology and microbiome studies. The Bray–Curtis dissimilarity algorithm was adopted to construct the sample distance matrix, and NMDS ordination was implemented via the vegan package (https://cran.r-project.org/web/packages/vegan/, accessed on 16 May 2024). The stress value was calculated to evaluate the goodness-of-fit of the final NMDS configuration, where a lower stress value indicates a better consistency between the original sample distance ranks and the low-dimensional ordination results. Permutational multivariate analysis of variance (PERMANOVA) was further conducted to statistically test the significance of intergroup separation based on the Bray–Curtis distance matrix to quantify whether microbial community structures differed significantly across predefined sample groups.

## 3. Results

### 3.1. General Structure of Adult Gut

**Figure 1:**
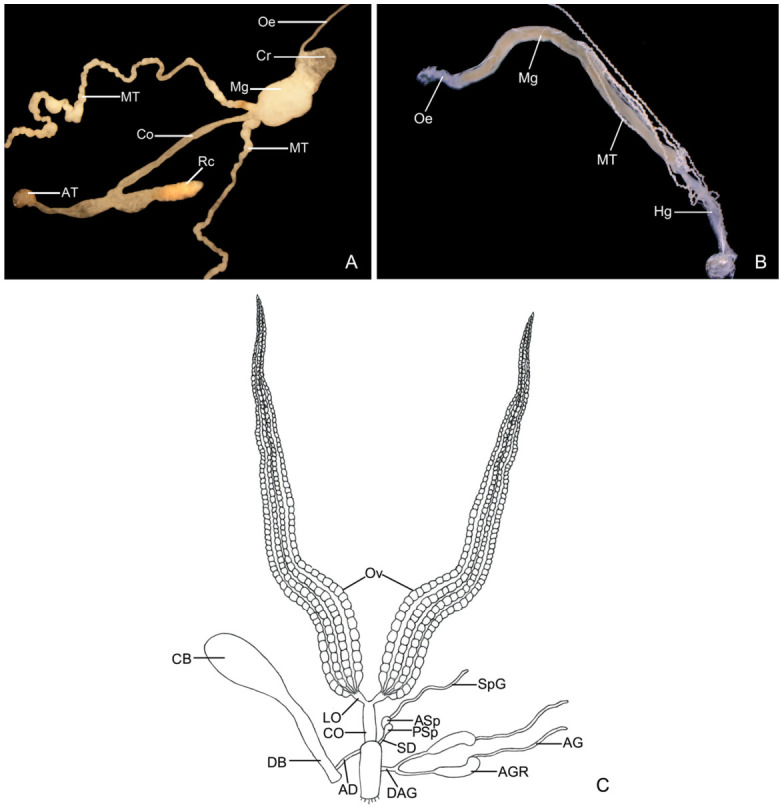
General structure of the alimentary canal and reproductive system of the striped stem borer, *Chilo suppressalis*. (**A**) Adult; (**B**) larva; (**C**) reproductive system of female. AD, afferent duct; AG, accessory gland; AGR, accessory gland reservoir; ASp, accessory spermatheca; AT, anal tube; CB, corpus bursae; Co, ileum; CO, common oviduct; Cr, crop; DAG, duct of accessory gland; DB, ductus bursae; LO, lateral oviduct; Mg, midgut; MT, Malpighian tubules; Oe, esophagus; Ov, ovary; PSp, principal spermatheca; Rc, rectum; SD, spermathecal duct; SpG, spermathecal gland. Scale bars: (**A**,**B**) = 0.4 mm; (**C**) = 0.5 mm.

The adult gut is structurally divided into the foregut, midgut, and hindgut. The foregut consists of a narrow esophagus and a bag-like crop; the esophagus extends posteriorly into the crop and continues into the anterior midgut. The pouch-shaped midgut is well-developed, originating from the crop to the hindgut. The hindgut comprises a long, convoluted ileum and a well-developed rectal pouch. Malpighian tubules emerge from the junction of the midgut and the ileum and run laterally along the midgut in the posterior direction. Upon reaching the anterior midgut, they follow their own course posteriorly and gather near the rectum (Figure 1A).

### 3.2. General Structure of Larval Gut

The gut of the larva is a continuous tube and is structurally divided into three distinct segments: the foregut, midgut, and hindgut. The foregut is slender and elongate, expanding posteriorly and constricted at its end. The midgut is well-developed and sac-like, originating from the posterior end of the foregut and extending to the anterior part of the hindgut. The hindgut is approximately one-third the length of the midgut and narrower in width. When freshly dissected, three segments exhibit distinct coloration, making them easily distinguishable: the foregut is translucent, the midgut is opaque and white, and the hindgut is yellowish-brown (Figure 1B).

### 3.3. General Structure of Female Reproductive System

The female reproductive system is complex and consists of the following characters (Figure 1C). An ovary (Ov) contains four ovarioles; the pedicel of each ovariole connects directly with a lateral oviduct (LO), and these ultimately connect with a common oviduct (CO). The basal region of the common oviduct enters a genital chamber (GC), which is laterally connected with a spermathecal duct (SD). The spermatheca consists of a principal spermatheca (PSp) and an accessory spermatheca (ASp); a convoluted spermathecal gland (SpG) emerges from the apex of the former. A duct of the accessory gland (DAG) emerges from the lower middle region of the genital chamber and apically bifurcates into a pair of accessory gland reservoirs (AGR). A coiled accessory gland (AG) is situated basally to the reservoir. An afferent duct (AD) emerges from the other side of the genital chamber, and its distal region connects with a ductus bursae (DB), which enlarges distally into a sac-like corpus bursae (CB).

### 3.4. Ultrastructure of the Midgut and Hindgut of Adult C. suppressalis

**Figure 2:**
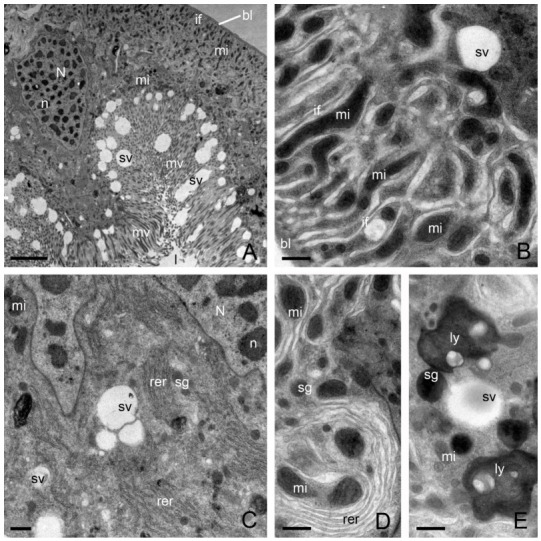
Electron micrographs of the first type of midgut cell in adult *C. suppressalis*. (**A**) Transverse section of the midgut. (**B**) High magnification of the basal region of midgut cells, showing well-developed basal infoldings containing abundant mitochondria and electron-lucent secretory granules. (**C**–**E**) High magnification of the middle region of midgut cells, showing nuclei and rough endoplasmic reticulum, secretory vesicles, and mitochondria scattered in the cytoplasm. bl, basal lamina; if, basal infoldings; l, lumen; ly, lysosome-like structures; Mi, mitochondria; n, nucleolus; N, nuclei; rer, endoplasmic reticulum; sg, secretory granules; sv, secretory vesicles. Scale bars: (**A**) 5.0 μm; (**B**–**E**) 0.5 μm.

**Figure 3:**
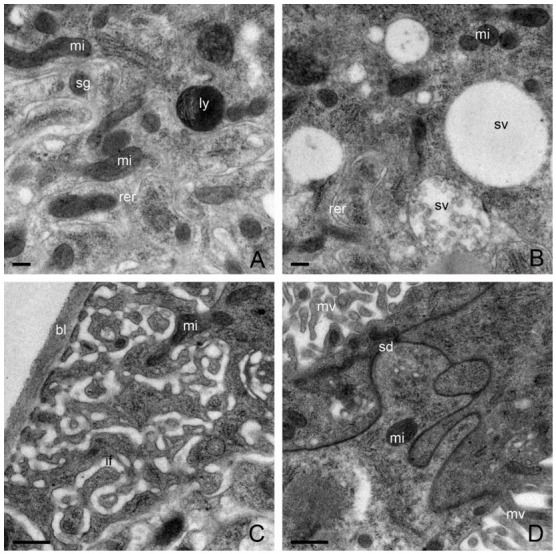
Electron micrographs of the midgut in *C. suppressalis*. (**A**,**B**) High magnification of the middle region of the first type of midgut cell. (**C**,**D**) High magnification of the middle region of the second type of midgut cell, showing scattered nuclei and rough endoplasmic reticulum, secretory vesicles, and mitochondria scattered in the cytoplasm. bl, basal lamina; if, basal infoldings; ly, lysosome-like structures; Mi, mitochondria; rer, rough endoplasmic reticulum; sd, septate desmosomes; sg, secretory granules; sv, secretory vesicles. Scale bars: (**A**,**B**) 0.2 μm; (**C**,**D**) 0.5 μm.

The midgut consists of two types of cells resting on a basal lamina (~0.5 μm thick). In the first type of cell, round or gourd-shaped nuclei with clumps of heterochromatin bounded by the nuclear membrane are found adjacent to the middle region (Figure 2A). Narrow, long, well-developed basal infoldings (~2.5 μm long) associated with elongated mitochondria are observed in the basal region (Figure 2A,B). Densely packed microvilli (~8.5 μm long) are obvious at the apical area (Figure 2A). Mitochondria occupy most of the cytoplasm, arranged in a row below the microvilli and orient with their long axes in the direction of the microvilli (Figure 2B). The cytoplasm contains extensive rough endoplasmic reticulum, electron-dense secretory granules, and abundant electron-lucent secretory vesicles (Figure 2C,D). These secretory vesicles vary in size and shape, and it appears that some small vesicles fuse to become larger ones (Figure 2C and Figure 3B). Many electron-dense, lysosome-like granules containing organelle debris are also observed in the cytoplasm (Figure 2E and Figure 3A).

**Figure 4:**
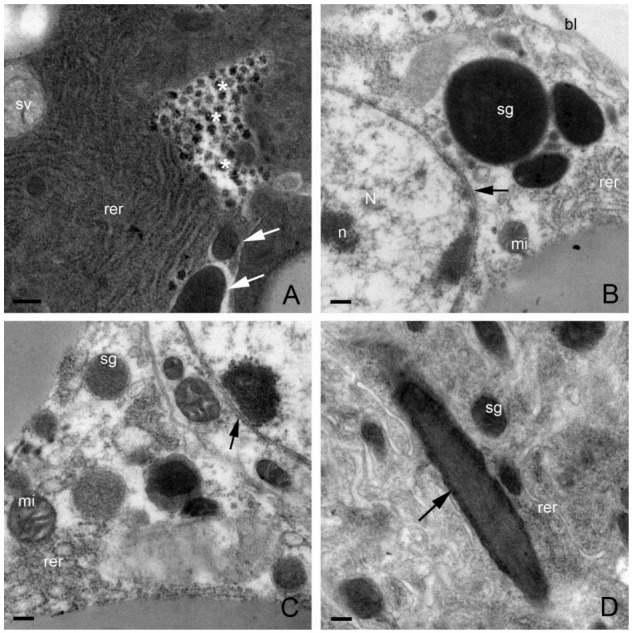
Electron micrographs of the second type of midgut cell in *C. suppressalis*. (**A**) Middle region of the first type of cell, showing well-developed rough endoplasmic reticulum, accumulations of glycogen granules (asterisks), and electron-lucent secretory vesicles. Arrows indicate microorganisms. (**B**,**C**) Close-up views of the nucleus, secretory granules, and mitochondria. Nuclei are bounded by the nuclear membrane (as indicated by arrows). (**D**) Close-up view of a microorganism. bl, basal lamina; Mi, mitochondria; n, nucleolus; N, nuclei; rer, rough endoplasmic reticulum; sg, secretory granules; sv, secretory vesicles. Microorganisms are arrowed in (**A**,**D**). Scale bars: (**A**–**D**) 0.2 μm.

In contrast, cells of the second type possessed very shallow basal labyrinths, though their apical border bears well-developed microvilli (Figure 3C). Heavy staining of the lateral cell membrane is observed in the cytoplasm (Figure 3D). Desmosomes cannot be discerned due to the over-staining of the membranes by osmium. Large areas of well-developed rough endoplasmic reticulum are found in the cytoplasm (Figure 4A). Accumulations of glycogen granules (~50.0 nm in diameter) and electron-lucent secretory vesicles are present in the cytoplasm (Figure 4A). These particles are spherical or possibly polyhedral. Electron-dense secretory granules and mitochondria with cristae are scattered throughout the cytoplasm (Figure 4B). Small secretory granules appear to fuse into larger ones. Nuclei with small nucleoli are bounded by the nuclear membrane (Figure 4B,C). The cytoplasm seems to be ruptured due to the load of virus-like particles. Electron-dense secretory granule and nuclei with small nucleoli are bounded by the nuclear membrane (Figure 4B,C). Microorganisms (~2.4 μm long, 0.2–0.5 μm in diameter) are scattered among the mitochondria (Figure 4A,D).

**Figure 5:**
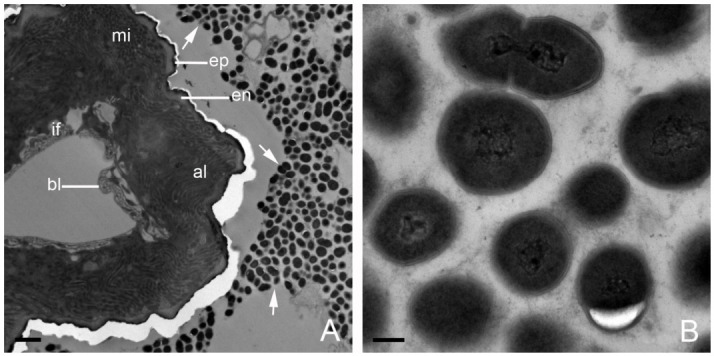
Electron micrographs of the hindgut of *C. suppressalis.* (**A**) Ileum cells contain abundant mitochondria. (**B**) Close-up of microorganisms in the lumen. al, apical leaflets; bl, basal lamina; en, endocuticle; ep, epicuticle; if, basal infoldings; Mi, mitochondria. Arrows indicate microorganisms in the lumen. Scale bars: (**A**) 2.0 μm; (**B**) 0.2 μm.

The ileum is lined with a highly convoluted cuticle containing an electron-dense epicuticle (0.5 μm thick) and an electron-lucent endocuticle (~0.3 μm thick) facing the lumen (Figure 5A). Cells beneath the cuticle are characterized by elaborated and extensive apical leaflets formed by invagination of the apical plasma membrane. Abundant mitochondria pack the cytoplasm and are associated with the leaflets. The basal plasma membrane invaginates into a few extremely wide and very shallow infoldings associated with mitochondria. Large numbers of oval or spherical microorganisms with an electron-dense membrane are observed in the lumen of this organ (Figure 5B). Within the membrane, electron-lucent vesicles and brush-like structures adjacent to the particles are also present (Figure 5B).

### 3.5. Ultrastructure of the Midgut and Hindgut of Larvae C. suppressalis

**Figure 6:**
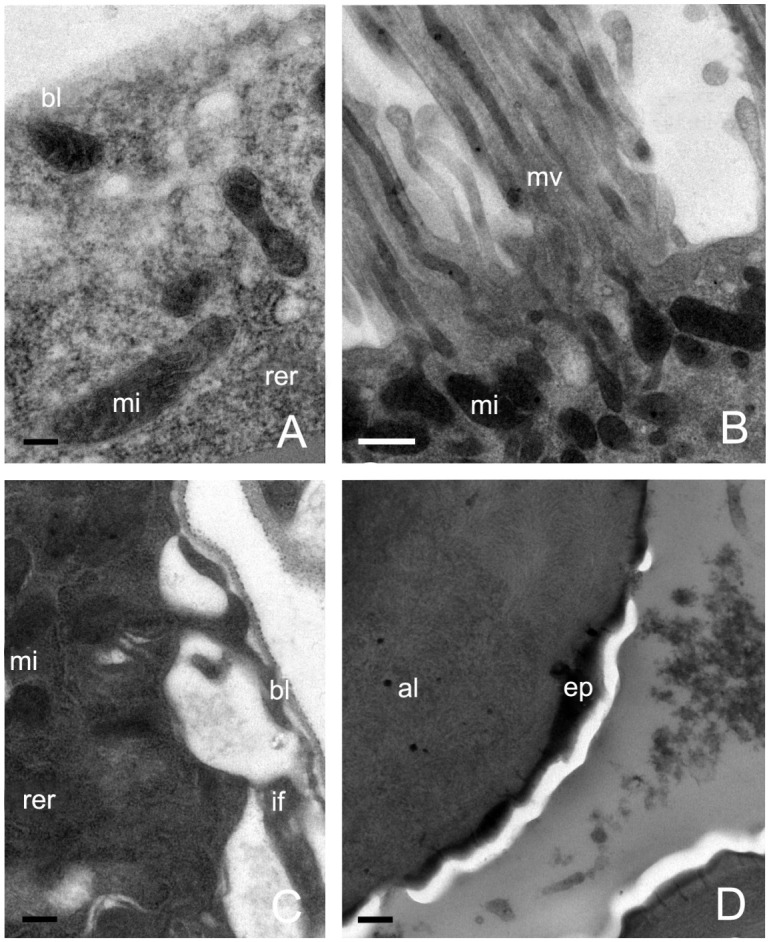
Electron micrographs of midgut cells of larvae of *C. suppressalis*. (**A**) Basal region of the cell. (**B**) Apical region of the cell. (**C**) Basal region of the hindgut cell. (**D**) Apical region of the hindgut cell. al, apical leaflets; bl, basal lamina; ep, epicuticle; if, basal infoldings; mi, mitochondria. mv, microvilli; rer, rough endoplasmic reticulum. Scale bars (**A**,**C**,**D**) 0.2 μm; (**B**) 0.5 μm.

The midgut cells rest on a basal lamina (~0.2 μm thick), and their basal region is equipped with elongated mitochondria with prominent cristae (Figure 6A). Rough endoplasmic reticulum scatters among the mitochondria. The apical plasma membrane bears well-developed apical microvilli (approximately 2.5–4.0 μm long), adjacent to which some oval mitochondria are observed (Figure 6B). The mitochondria and rough endoplasmic reticulum occupy most of the cytoplasm.

The hindgut cells rest on a layer of very thin basal lamina (~0.05 μm thick) (Figure 6C). The basal plasma membrane invaginates into very wide infoldings associated with oval mitochondria and rough endoplasmic reticulum, whereas the apical plasma membrane is elaborated into well-developed apical leaflets (Figure 6D).

### 3.6. Ultrastructure of Ovary of Female C. suppressalis

**Figure 7:**
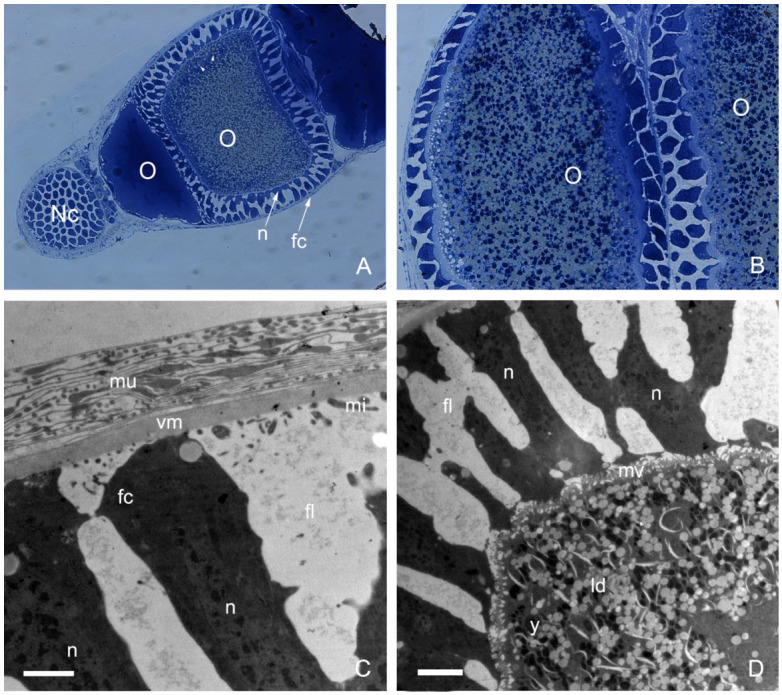
Electron micrographs of the ovary of female *C. suppressalis*. (**A**) Cylindrical follicle cells of the ovary. Arrowheads indicate lipid droplets. (**B**) Magnification of the follicle cells.(**C**) Basal region of the oocyte.(**D**) Close-up of the nurse cell. fc, follicle cell; fl, infoldings; ld, lipid droplets; mi, mitochondria; mu, muscles; n, nucleus; Nc, nurse cell; o, oocyte; vm, vitelline membrane; y, yolk granules. Scale bars: (**C**) 2.0 μm; (**D**) 5.0 μm.

**Figure 8:**
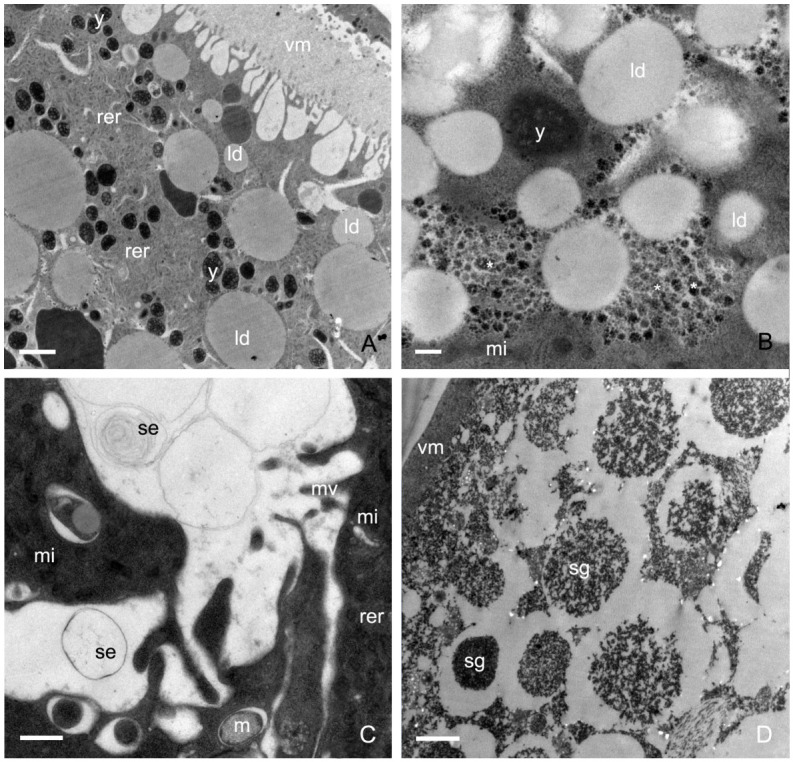
Electron micrographs of the ovary of female *C. suppressalis*. (**A**) The basal region of the oocyte cell. (**B**,**C**) The middle region of the cell. Asterisks indicate virus-like particles scattered in the cytoplasm. (**D**) The basal region of the nurse cell. ld, lipid droplets; mi, mitochondria; rer, rough endoplasmic reticulum; se, secretions; sg, secretory granules; y, yolk granules. Scale bars: (**A**,**D**) 2.0 μm; (**B**) 0.2 μm; (**C**) 0.5 μm.

The ovary contains numerous nurse cells. Cylindrical follicle cells with evident nuclei entirely envelop the oocyte (Figure 7A,B). The basal membrane of the follicle cells invaginates into long, wide infoldings associated with elongated or oval mitochondria (Figure 7C). Well-developed microvilli are visible at the apical area of the follicle cells, and the cytoplasm of these cells is filled with yolk granules and lipid droplets (Figure 7A,D and Figure 8A). These granules and droplets vary in size and electron density, and some smaller ones fuse to form larger ones (Figure 8A). Rough endoplasmic reticulum and virus-like particles are scattered among the granules and droplets (Figure 8B). Abundant elongated or oval mitochondria of various sizes are visible throughout the cytoplasm. Sparse, less-developed apical microvilli and numerous elongated mitochondria appear to participate in secretion (Figure 8C). Unlike the oocyte, the nurse cell is filled with secretory granules of different sizes and electron densities (Figure 8D).

Given the structural and ultrastructural differences in the gut and ovary across developmental stages, we next characterized the associated microbiota to explore how these anatomical features might correlate with microbial community composition and potential transmission routes.

### 3.7. Analysis of Bacterial 16S rDNA Gene Sequences

A total of 687,799 sequences clustering into 2524 operational taxonomic units (OTUs) are generated to analyze microbiota (Table 1). Detected OTU numbers, as well as Chao and Shannon indices, are calculated as alpha diversity indicators. The relative sequence abundance of 17 phyla differs significantly among the gut, egg and ovary (Kruskal–Wallis test, *p* < 0.0001) (Table S1). The bacteria identified in the larval gut (JMG) are more diverse than those in the adult egg (JEG) (Table 1).

### 3.8. Diversity of Gut Microbiota

**Figure 9:**
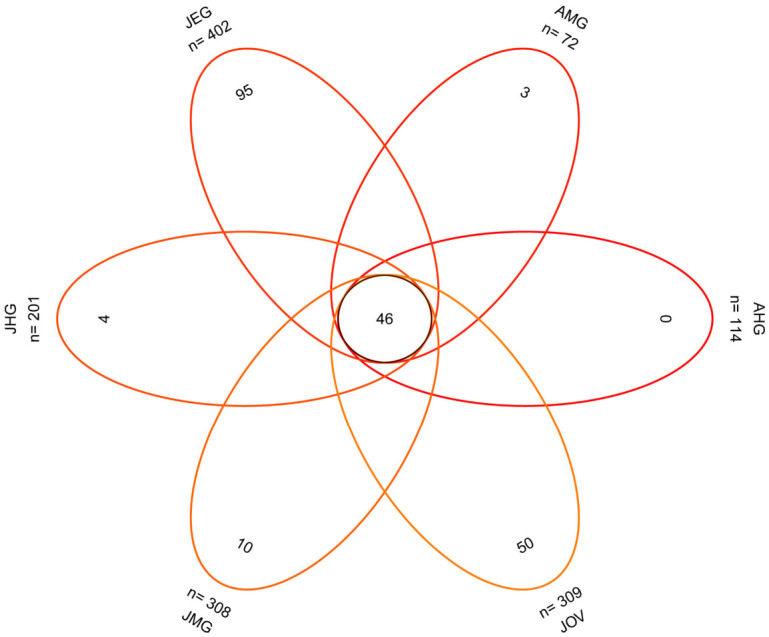
Venn diagram showing OTUs unique to each sample and those common to two or more samples. AHG, hindguts of adult; AMG, midguts of adults; JHG, hindguts of larvae; JEG, eggs laid by females; JMG, midguts of larvae; JOV, ovary of females.

A total of 46 OTUs are shared by all samples (gut, ovary, and egg) (Figure 9), and the core OTUs belong to the following four phyla: Proteobacteria, Bacteroidetes, Firmicutes, and Actinobacteria (Figure S1). The OTUs detected from different samples are grouped into distinct families, with varying relative sequence abundances: those from the midgut comprise 85 families, with Enterobacteriaceae (35.93–94.12%) and Enterococcaceae (3.86–12.41%) being the most abundant families (Tables S2A and S3); those from the hindgut are classified into 71 families, with Enterobacteriaceae (67.25–77.89%) and Enterococcaceae (10.4–11.4%) as the predominant families (Tables S2B and S3); those identified from the egg are classified to 101 families, with Bacillaceae (32.5%), Streptococcaceae (16.2%), Enterococcaceae (8.3%), Enterobacteriaceae (5.3%), and Flavobacteriaceae (5.1%) being the prevalent families (Table S3); and those from the ovary are grouped into 96 families, with Enterobacteriaceae (40.7%), Enterococcaceae (28.8%), and Bacillaceae (13.1%) as the predominant families (Table S3).

OTUs from the different gut compartments show great variation between larvae and adults. OTUs from the adult hindgut (AHG) are grouped into 45 families, with Enterobacteriaceae (78.4%), Enterococcaceae (11.5%), and Bacillaceae (4.0%) being the predominant ones (Table S3). OTUs from the adult midgut (AMG) are classified into 35 families, of which Enterobacteriaceae (94.1%) is the dominant taxa (Table S3). In comparison, OTUs from the JHG are grouped into 67 families, with Enterobacteriaceae (66.7%), Bacillaceae (11.3%), Enterococcaceae (10.1%), and Streptococcaceae (5.9%) being the prevalent families (Table S3). OTUs from the larval midgut (JMG) are classified into 84 families, and Enterobacteriaceae (35.9%), Bacillaceae (26.9%), Streptococcaceae (14.1%), Enterococcaceae (12.4%), and Halomonadaceae (4.9%) are the predominant families (Table S3).

### 3.9. Taxonomic Distribution of Gut Microbiota of C. suppressalis

**Figure 10:**
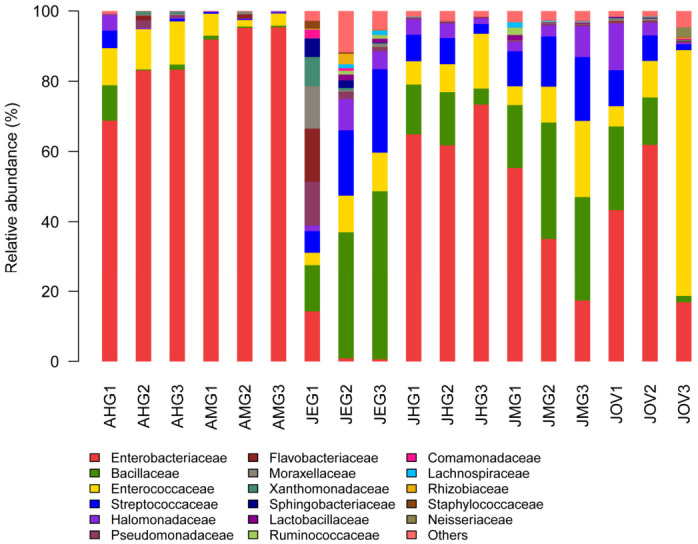
Composition of gut microbiota at the family level across different developmental stages of *C. suppressalis*. The *Y*-axis represents the proportion of each taxon. Abbreviations: AHG1–AHG3, hindguts of adults; AMG1–AMG3, midguts of adults; JEG1–JEG3, eggs oviposited by females; JHG1–JHG3, hindguts of larvae; JMG1–JMG3, midguts of larvae; JOV1–JOV3, ovaries of females.

**Figure 11:**
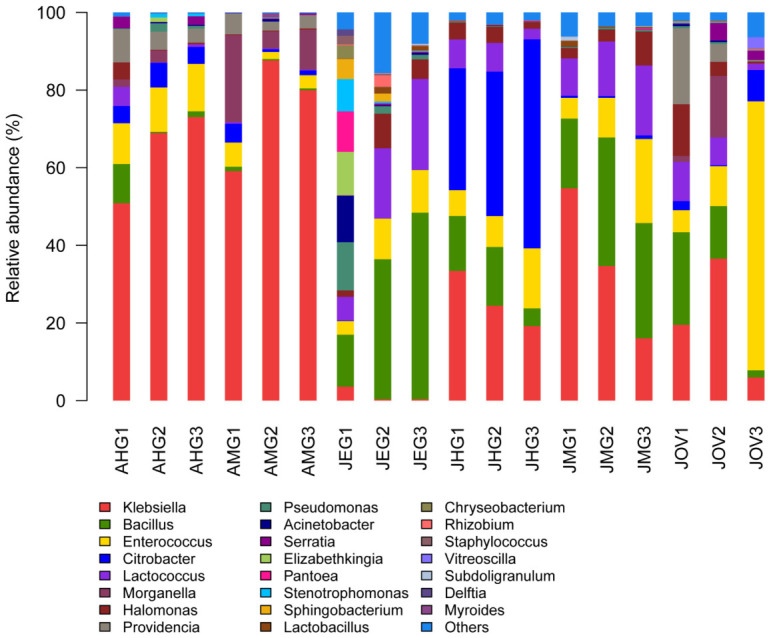
Compositions of the gut microbiota at the genus level across different developmental stages of *C. suppressalis*. The composition of each sample is based on the taxonomic assignment of the 16S rDNA sequences. The *Y*-axis represents the proportion of each taxon. Abbreviations: AHG1–AHG3, hindguts of adults; AMG1–AMG3, midguts of adults; JEG1–JEG3, eggs oviposited by females; JHG1–JHG3, hindguts of larvae; JMG1–JMG3, midguts of larvae; JOV1–JOV3, ovary of females.

At the phylum level, a homogeneous distribution is observed in all samples (AHG, AMG, JMG, JEG, JHG JOV), dominated by Proteobacteria (8.6–96.3%) and Firmicutes (2.5–89.7%), respectively (Figure S1; Table S4). The two predominant phyla, Proteobacteria and Firmicutes, show significant changes between JEG and JMG (Figure 10 and Figure S1; Tables S3 and S4). A total of 17 bacterial phyla are grouped into 129 families, with Enterobacteriaceae being the most predominant family, followed by Bacillaceae and Enterococcaceae (Table S3). The families Bacillaceae, Enterobacteriaceae, Enterococcaceae, Halomonadaceae, Moraxellaceae, Streptococcaceae, and Pseudomonadaceae are found across all samples (AHG, AMG, JHG, JMG, JEG, JOV), although they exhibit different relative sequence abundances. In contrast, Brucellaceae, Clostridiaceae, Lactobacillaceae, Microbacteriaceae, and Planococcaceae are only found in JEG, JOV, JHG, and JMG, despite their low relative sequence abundances (Figure 10 and Figure 11; Table S3). Bacillaceae is enriched in the egg (32.5%), followed by JMG (26.9%) and JOV (13.1%). Enterobacteriaceae is enriched in AMG (94.1%), AHG (78.4%), and JHG (66.7%). Enterococcaceae predominates in JEG (28.8%), JMG (12.41%), and AHG (11.5%). Halomonadaceae and Streptococcaceae are abundant in JOV (5.8%, 6.4%), JEG (5.2%, 16.2%), and JMG (4.9%, 14.1%), respectively. Moraxellaceae is enriched in JEG (4.4%) and presents lower relative sequence abundances in JOV (0.4%), AHG (0.2%), and JMG (0.2%). Some families have much lower relative sequence abundances; for example, Brucellaceae in JEG (0.4%) and JOV (0.2%); Clostridiaceae and Lactobacillaceae in JMG (0.2%, 0.7%) and JEG (0.2%, 1.0%); Microbacteriaceae and Planococcaceae in JEG (0.2%); and Pseudomonadaceae in JOV (0.4) and JMG (0.4%). These bacteria exhibit variation in relative sequence abundance associated with developmental stage, diet, and gut compartment.

Based on the analysis of developmental stage and gut compartments, the bacterial genera from adults and larvae show distinct distributions (Figure 11; Table S5). *Klebsiella* is dominant in AMG (64.3%) and AHG (75.6%), followed by *Enterococcus* in AHG (11.4%) and *Morganella* in AMG (12.4%). *Citrobacter* and *Enterococcus* in AHG (5.1%, 11.4%) exhibit higher relative sequence abundances than those in AMG (2.2%, 3.8%). In JEG, *Bacillus* (32.5%) prevails, followed by *Lactococcus* (15.8%), *Enterococcus* (8.3%), and *Halomonas* (5.2%). *Citrobacter* is dominant in the JHG (40.81%), followed by *Klebsiella* (25.7%). *Klebsiella* (35.1%) is dominant in JMG, followed by *Bacillus*, *Lactococcus*, and *Enterococcus* (26.9%, 13.9%, 12.4%). *Halomonas* is present in JHG and JMG with lower relative sequence abundances (3.4%, 4.9%). *Enterococcus* is notably dominant in JOV (28.4%), followed by *Klebsiella* (28.4%), *Bacillus* (13.1%), and *Lactococcus* (6.3%); *Halomona* and *Morganella* are present with similar abundances (5.8% each). *Providencia* is only identified in AMG, AHG, and JOV (3.7%, 5.6%, and 8.2%, respectively).

### 3.10. Development-, Compartment- and Diet-Related Variations in the Microbiota

**Figure 12:**
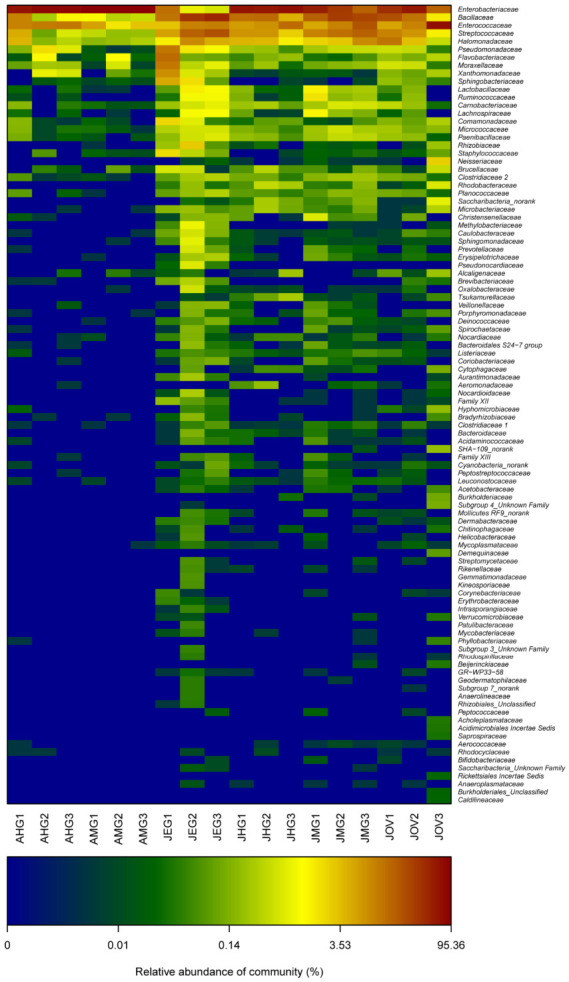
Relative sequence abundance and composition of gut microbiota in all samples. The heatmap represents the proportions of OTUs at the family level. The X-coordinate represents the sample of each population, and the Y-coordinate represents the taxon. The color code indicates relative sequence abundance, ranging from blue (low relative sequence abundance) through yellow to red (high relative sequence abundance). Abbreviations: AHG1–AHG3, hindguts of adults; AMG1–AMG3, midguts of adults; JEG1–JEG3, eggs oviposited by females; JHG1–JHG3, hindguts of larvae; JMG1–JMG3, midguts of larvae; JOV1–JOV3, ovary of females.

In *C. suppressalis*, 99 bacterial genera are identified. The influence of gut compartment proves to be significant, with well-defined clusters forming within larvae (i.e., JHG, JMG) and adults (i.e., AHG, AMG). Bacteria from the JOV and JEG are more heterogeneous in terms of cluster formation (Figure 12). Enterobacteriaceae, Bacillaceae, and Streptococcaceae exhibit significant differences in relative sequence abundance among the gut compartments. Enterobacteriaceae is dominant in the AHG and AMG (78.4%, 94.1%) but decreases in the JHG and JMG (66.7%, 35.9%). In contrast, Bacillaceae is less abundant in the AHG and AMG (4.0%, 0.7%) but increases significantly in the two larval gut regions (11.3%, 26.9%). Streptococcaceae and Halomonadaceae are less abundant in the AHG (1.9%, 1.6%) and AMG (0.3%, 0.3%) but increase in the JHG (5.9%, 3.4%) and JMG (14.1%, 4.9%) (Figure 12; Table S3).

**Figure 13:**
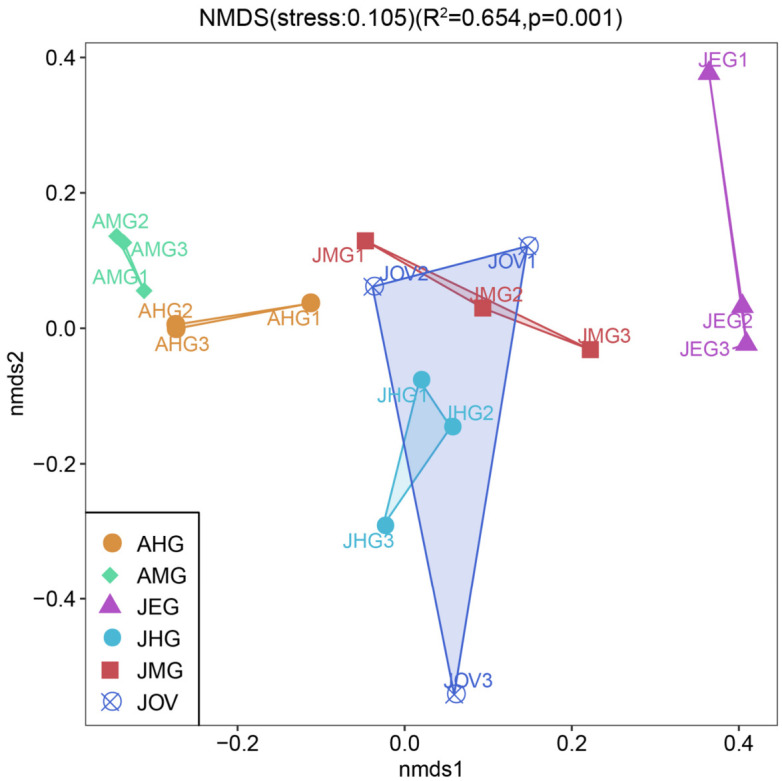
NMDS of the gut, ovary and egg microbiota of *C. suppressalis*. The samples are clustered by developmental stage, diet, and compartment and are represented with different colors and shapes: The ellipses represent the standard error of the centroid for each group of samples with a confident limit of 95%. Abbreviations: AHG1–AHG3, hindguts of adults; AMG1–AMG3, midguts of adults; JEG1–JEG3, eggs oviposited by females; JHG1–JHG3, hindguts of larvae; JMG1–JMG3, midguts of larvae; JOV1–JOV3, ovaries of females.

The non-metric multidimensional scaling (NMDS) analysis reveals a clear separation of all samples according to developmental stage and gut regions, as well as a closer association between JMG and JOV. Clusters are well-defined, with relatively great variability observed in the samples (i.e., JMG, JHG, AMG, AHG, EGG), except for JOV. The JEG cluster exhibits the most distinct microbiota, followed by the AHG and AMG clusters; the JHG cluster shows an intermediate composition. The JEG and JOV clusters exhibit higher inter-sample variation. The JHG cluster shows an intermediate composition with respect to AMG, AHG, and JHG clusters. The JMG and JOV clusters are similarly homogeneous; the JEG is the most heterogeneous, followed by JOV (Figure 13).

## 4. Discussion

### 4.1. Morphology and Ultrastructure of Gut in C. suppressalis

In the present study, three types of basal infoldings were observed in the gut cells of *C. suppressalis*: (1) very narrow, long infoldings; (2) shallow, scattered infoldings; and (3) extremely wide, very shallow infoldings. The first type of infolding was frequently reported in many insects, such as the filter chamber of leafhopper *Cicadella viridis* and midgut of midge *Belgica antarctica* [1,25]; the second type of infolding was morphologically similar to those previously found in the midgut of the leafworm *Alabama argillacea* [26], the ileum of *C. viridis* and the rectum of the hangingfly *Bittacus cirratus* [1,27]. Well-developed apical microvilli only observed in the midgut of *C. suppressalis* were similar to those reported in the same region of katydids *Gampsocleis gratiosa* and the black soldier fly *Hermetia illucens* [28,29]. The microvilli and infoldings can enormously increase the surface area of the cell membrane and are generally considered to indicate a secretory or ion-transport function [30]. Some investigators have suggested that such structures provide “effective coupling of passive solvent movements to active salt transport during fluid” [31,32]. Well-developed infoldings, microvilli, abundant mitochondria, extensive rough endoplasmic reticulum and many secretory vesicles observed in the cytoplasm of *C. suppressalis* are likely to suggest a potential role in ion transport, and this needs to be investigated further.

Lysosome-like granules were observed in the midgut epithelium of *C. suppressalis*, resembling those in the midgut of the cockroach *Periplaneta americana* [33] and *G. gratiosa* [28]. Since such structures are involved in the ultrastructural changes via phagocytic or autophagic activity, this suggests that the midgut cells of *C. suppressalis* are probably in an aging stage and undergoing an ultrastructural alteration. The most striking character of ileum cells in *C. suppressalis* was well-developed apical leaflets associated with abundant mitochondria, resembling those found in the *C. viridis* and the fruit fly *Bactrocera dorsalis* [1,34]. Such leaflets can greatly increase the contact surface with the luminal fluid [35], and it has been proposed that “apical infoldings and their associated mitochondria form mitochondrial pumps for ion transport from the gut lumen to the hemocoel” [36]. Thus, the extensive apical leaflets in the hindgut cells of *C. suppressalis* might play a role in ion reabsorption.

### 4.2. Morphology and Ultrastructure of the Ovary of C. suppressalis

The female reproductive system of *C. suppressalis* was similar to other lepidopterans, such as *Psilogramma menephron*, *Tirathaba rufivena*, *Holcocerus hippophaecolus*, *Opisina arenosella* [37,38,39,40], and they shared the following characters: (1) it consisted of paired ovaries, each containing three to five ovarioles; (2) two lateral oviducts fused into a common oviduct; (3) a genital chamber connected laterally with a long, convoluted spermatheca gland; and (4) a spermatheca composed of a primary gland and an accessory gland. However, some differences exist among species, such as the moths *Grapholita molesta*, *Phauda flammans*, species of the genus *Palumbina*, *Sphecodoptera sheni* and the butterfly *Tirumala limniace* [7,41,42,43,44].

Follicle cells play an important role in egg formation, and margins of the follicular cells were blurred at the beginning of oogenesis [45]. The distribution of abundant mitochondria and rough endoplasmic reticulum was very similar to that observed in the bug *Lygus lineloaris* [46], possibly suggesting that the follicle cells of *C. suppressalis* require substances from the hemolymph to synthetize egg precursors. In the early stage of eggshell formation, follicular cells begin to secrete and form proteinaceous yolk membrane [45]. With the gradual densification of the yolk membrane, follicular cells may secrete a dense inner eggshell. Subsequently, secretions continue to accumulate on the inner eggshell to form the outer eggshell.

### 4.3. Microbiota Associated with Gut, Ovary and Egg

In the present study, we characterized the microbiota composition and relative sequence abundance of *C. suppressalis* across its life stages. The phyla Proteobacteria, Firmicutes, Bacteroidetes, and Actinobacteria together constituted the majority of the microbiota (90.7–99.9%), although their relative abundances exhibit considerable variation. Proteobacteria has been reported to be involved in nitrogen [47] and carbohydrate degradation, such as starches and hemicellulose [48]. Firmicutes has been suggested to participate in energy absorption from the diet and may influence insect development [49]. Bacteroidetes promoted lignocellulose breakdown in anaerobic environments, resulting in the release of sugars and volatile fatty acids [50]. Actinobacteria produces cellulase, xylanases and lignases that facilitate the degradation of lignocellulosic substances in wood-feeding organisms [51] or aid in lipid digestion in grain-feeding beetles [52]. Bacterial diversity was notably greater in the larval midgut of *C. suppressalis* compared with other regions or eggs. Since the midgut is the main region of food digestion, its pH, physiological habitat and available food may facilitate the residence of abundant microbiota.

The recurrent detection of these core genera in the ovary, egg and gut indicates a partial overlap in bacterial composition among these compartments. *Klebsiella* has been suggested to contribute to nitrogen requirements [25,53]; *Enterococcus* has been reported to be associated with insecticide and pathogen resistance in other insect species [22,54]; and *Bacillus* is a major bacterial group for producing cellulases and proteases, so as to enhance digestion and absorption [55]. *Lactococcus* and Enterococcus influence the degradation of free amino acids [56]; *Citrobacter* in the gut produce proteases involved in proteolysis [3]. Whether these bacteria are capable of surviving, replicating, or exerting any biological function within these organs remains unknown and merits further investigation.

Diet and developmental stage are known to modulate microbiota in many insects [57,58,59,60], and some bacteria may suppress other phyla in the same habitat [61]. The potential symbiont or beneficial bacteria may have evolved in close association with their hosts or was simply transient in the gut from food and may be able to colonize in different gut regions [62,63]. The diets of adults and larvae of *C. suppressalis* are markedly different in nutritional ingredients, and the pulps ingested by larvae contain some secondary compounds (i.e., oxalic acid and tannin). Substantial variation in microbiota composition among AMG, JMG and JEG may be attributed to a combination of metamorphosis and diet, as holometabolous insects typically empty their gut contents and undergo gut remodeling during pupation. After emergence, adult feeding might stimulate the growth of persisting bacteria, or new bacterial taxa derived from the diet could restore community richness. Further research is necessary to elucidate the potential functions of the core bacteria identified in *C. suppressalis*. Our findings suggest that the rapid fluctuation of microbiota from larval to adult guts is probably influenced by dietary shift and microbial community reassembly, factors known to modulate microbiota as previously indicated in other Lepidopterans, Coleopterans, and Isopterans [58,61,64,65,66,67]. Further investigations involving culture-based methods, fluorescence in situ hybridization (FISH), and gnotobiotic experiments are required to examine whether these bacteria are genuinely transmitted and whether they have any impact on host biology.

## 5. Conclusions

In summary, our study revealed that the larval midgut harbored the highest bacterial diversity. The predominant phyla were Proteobacteria and Firmicutes, and the dominant families were Enterobacteriaceae, followed by Bacillaceae and Enterococcaceae. Diet, developmental stage, and gut compartment likely contributed to the high genus-level diversity observed in both larvae and adults. These findings provide important insights into insect–bacteria symbioses and may offer a microbiota-based perspective for the future control of lepidopteran pests.

## Figures and Tables

**Table 1:** Diversity of gut bacterial communities based on sequencing.

Developmental Stages	Diet Type	Sample	Abbreviations	Reads	Bases (bp)	OTUs	Coverage	Richness Estimate		Diversity Index
								Chao1	S	Shannon
Larvae/Adults	Pulps	Midgut	JMG1	40,138	17,434,180	248	0.998729	303	40,769	1.82
			JMG2	30,164	13,022,139	156	0.998110	225	30,361	1.76
			JMG3	38,225	16,566,823	185	0.998483	242	38,626	2.03
		Hindgut	JHG1	39,198	17,058,995	154	0.998954	182	39,778	1.78
			JHG2	29,999	13,129,341	97	0.999033	142	30,626	1.84
			JHG3	41,964	18,898,628	105	0.999261	129	44,068	1.45
	No	Midgut	AMG1	29,622	13,457,815	116	0.999122	134	31,413	1.29
			AMG2	30,500	13,485,925	117	0.999512	138	32,143	1.30
			AMG3	31,377	14,267,149	119	0.999076	140	33,296	1.31
		Hindgut	AHG1	42,226	18,171,436	94	0.999124	145	42,360	1.65
			AHG2	40,005	17,192,557	137	0.998700	178	40,081	0.78
			AHG3	36,054	15,537,581	100	0.998835	151	36,219	1.35
Ovary/Egg	-	Ovary	JOV1	38,043	16,419,021	60	0.999448	95	38,220	0.86
			JOV2	42,406	18,247,431	77	0.999245	148	42,501	1.00
			JOV3	37,306	16,100,611	66	0.999383	94	37,483	0.89
		Egg	JEG1	30,535	14,170,587	102	0.999247	115	33,125	1.85
			JEG2	41,371	18,295,563	106	0.999202	150	42,683	1.68
			JEG3	35,379	16,197,473	117	0.998954	159	37,890	1.94

Note: JMG1–JMG3, larval midgut; JHG1–JHG3, larval hindgut; AMG1–AMG3, adult midgut; AHG1–AHG3, adult hindgut; JOV1–JOV3, adult ovary; JEG1–JEG3, adult egg.

## Data Availability

The raw reads were deposited into the National Center for Biotechnology Information (NCBI) Sequence Read Archive (SRA) database under the BioProject: PRJNA1142714. https://dataview.ncbi.nlm.nih.gov/object/PRJNA1142714?archive=sra (accessed on 4 May 2025).

## Supplementary Materials

The following supporting information can be downloaded at: https://www.mdpi.com/article/10.3390/insects17070682/s1, Figure S1. Bacterial composition (phylum level) of the microbiota along the midgut, hindgut, ovary and egg of *Chilo suppressalis*; Table S1. Relative bacterial abundance belonging to 18 phyla across all samples; Table S2A. OUT name of bacteria of all midgut samples in venn; Table S2B. OUT name of bacteria of all hindgut samples in venn; Table S2C. The OTUs coming from the ovary and egg in venn; Table S3. Mcrobial community percent at family level (unmerged biological replicates); Table S4. Mcrobial community percent at phylum level; Table S5. Mcrobial community percent at genus level.

## Abbreviations

AHG: Hindgut of adult; AMG: Midgut of adult; JEG: Egg deposited by female; JHG: Hindgut of larvae; JMG: Midgut of larvae; JOV: Ovary of female; NCBI: National Center for Biotechnology Information; NMDS: Non-metric multidimensional scaling; OTUs: Operational Units; PCR: Polymerase chain reaction; SSB: Striped stem borer; SRA: Sequence Read Archive.
