# Supplementary material for: Structure of the Gut and Ovary, with Associated Microbiota Across Life Stages in the Striped Stem Borer Chilo suppressalis (Lepidoptera: Crambidae)
Source: Insects. 2026 Jun 30;17(7):682. doi: 10.3390/insects17070682 (PMC13411576; doi:10.3390/insects17070682)
Supplement: Supplementary file 1 [file insects-17-00682-s001.zip › Supporting Information--Figure legends and Table titles (25, 2026).pdf]

Supplementary Information (Figure S1, Tables S1-S5)

Structure of gut and ovary, with associated bacteria across life stages in the striped stem borer *Chilo suppressalis* (Lepidoptera: Crambidae)

Haiying Zhong, Fang Li, Kaili Yu and Juefeng Zhang*

Institute of Plant Protection and Microbiology, Zhejiang Academy of Agricultural Sciences; Hangzhou 310021, China.

*Corresponding: zhangjuefeng@sina.com

Running title: Gut and ovary and associated bacteria of *Chilo suppressalis*

Supplementary Information:

Figure S1. Bacterial composition (phylum level) of the microbiota along the midgut, hindgut, ovary and egg of *Chilo suppressalis*.

Table S1. Relative bacterial abundance belonging to 18 phyla across all samples.

Table S2A. OUT name of bacteria of all midgut samples in venn.

Table S2B. OUT name of bacteria of all hindgut samples in venn.

Table S2C. The OTUs coming from the ovary and egg in venn.

Table S3. Mcrobial community percent at family level (unmerged biological replicates).

Table S4. Mcrobial community percent at phylum level.

Table S5. Mcrobial community percent at genus level.
