# Supplementary figures and images for: Structure of the Gut and Ovary, with Associated Microbiota Across Life Stages in the Striped Stem Borer Chilo suppressalis (Lepidoptera: Crambidae)
Source: Insects. 2026 Jun 30;17(7):682. doi: 10.3390/insects17070682 (PMC13411576; doi:10.3390/insects17070682)

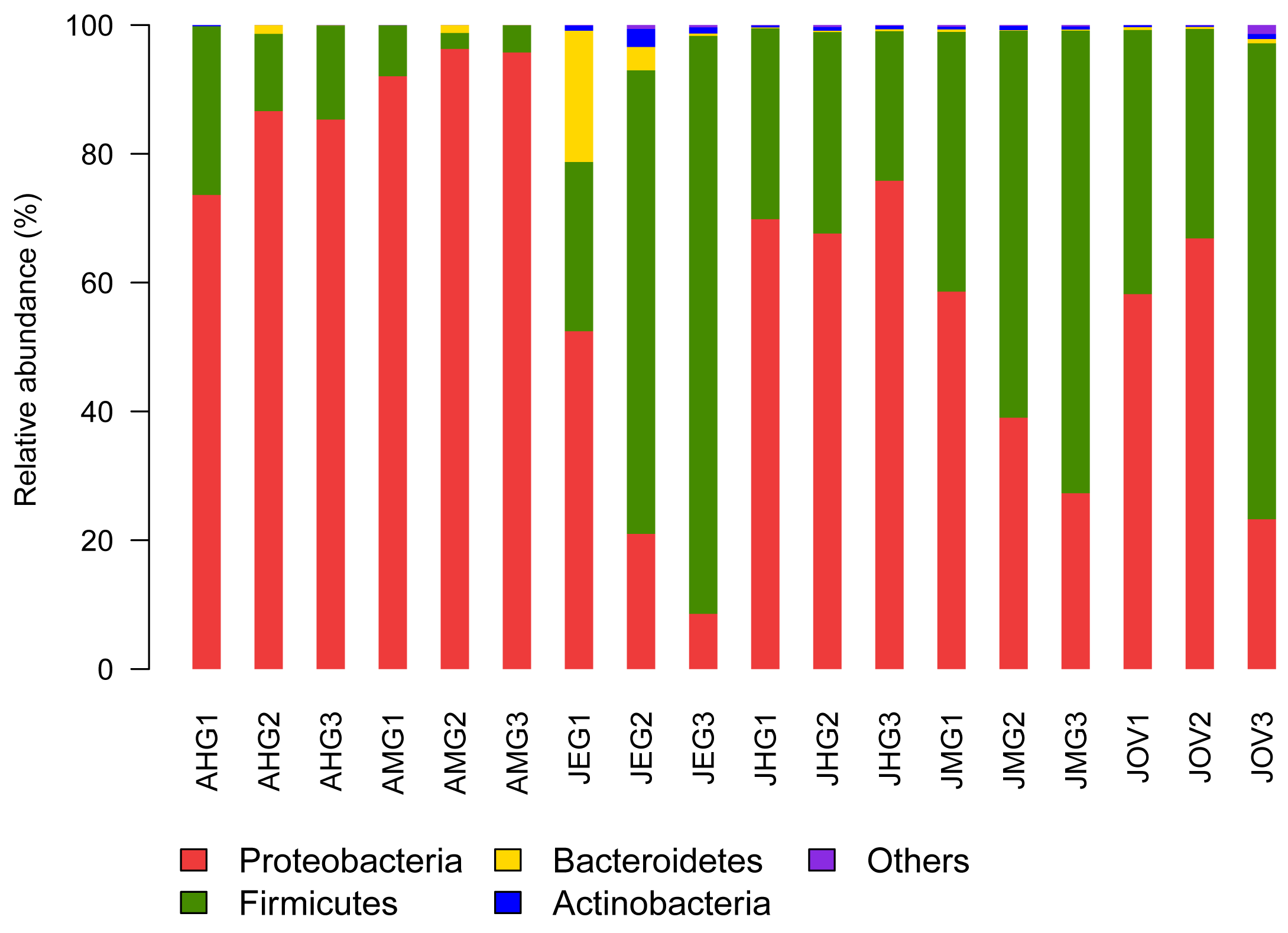

Supplement: Supplementary file 1 [file insects-17-00682-s001.zip › Figure S1.tif]
